# Rheumatoid arthritis patients with fibromyalgic clinical features have significantly less synovitis as defined by power Doppler ultrasound

**DOI:** 10.1186/s12891-016-1258-6

**Published:** 2016-09-23

**Authors:** Aneela N. Mian, Khaldoun Chaabo, Julekha Wajed, Sujith Subesinghe, Nicola J. Gullick, Bruce Kirkham, Toby Garrood

**Affiliations:** 1 Department of Rheumatology, Guys and St Thomas’ NHS Trust, Great Maze Pond, London, SE1 9RT UK; 2 Department of Rheumatology, King’s College Hospital London, Denmark Hill, SE5 9RS UK; 3 Department of Academic Rheumatology, King’s College London, 10 Cutcombe Road, London, SE5 9RT UK

**Keywords:** Rheumatoid arthritis, Fibromyalgic rheumatoid, Ultrasound

## Abstract

**Background:**

In patients with rheumatoid arthritis (RA) clinical measures of disease activity may not reliably discriminate between patients with active inflammatory disease and those with concomitant fibromyalgia (FM). Recent work has shown RA patients with a 28 tender joint count (TJC) minus swollen joint count (SJC) of 7 or more (joint count criteria) are more likely to meet classification criteria for FM. This study aimed to determine whether RA patients meeting clinical criteria for FM had lower levels of joint inflammation as determined by ultrasound (US).

**Methods:**

RA patients with DAS28 > 2.6 were recruited. Patients underwent clinical assessment including ultrasound examination of the hands and wrists with quantification of grey scale (GS) and power Doppler (PD) synovitis. Patients completed questionnaires to assess pain, fatigue, disability and psychological comorbidity.

**Results:**

Patients meeting either of the FM criteria had higher scores for disease activity, depression, disability and fatigue. Those meeting both the joint count and classification FM criteria had significantly lower levels of GS and PD inflammation on US.

**Conclusions:**

RA patients with concomitant FM, as determined by widespread soft tissue tenderness but fewer clinically inflamed joints, have higher disease activity scores but may have lower levels of synovial inflammation on US. This has implications for the identification and management of these patients who may not respond to conventional therapy and hence be more suitable for alternative approaches to treatment.

## Background

Rheumatoid Arthritis (RA) is a chronic inflammatory arthritis which, if untreated, can lead to progressive joint damage, disability, and reduced quality of life. Evidence suggests that the a ‘treat to target’ approach achieves better outcomes [1]. The frequently used twenty-eight joint disease activity score (DAS28) includes both objective and subjective measures and hence non-inflammatory factors contribute to patient-reported measures, pain and tenderness.

Fibromyalgia (FM) is a condition characterized by chronic widespread pain and tender points on clinical examination. In the majority of patients, it is associated with psychological comorbidity, sleep disturbance, fatigue and other somatic symptoms which are reflected in recently proposed diagnostic criteria [2]. An estimated 20 % of patients with RA have co-existing fibromyalgia [3]. The presence of concomitant FM can make treatment decisions challenging, as disease activity scores can be high despite limited clinical evidence of active synovitis [4]. As the DAS28 cannot distinguish these two groups of patients who require different treatment pathways, it is important to ascertain other discriminatory measures. It is unknown whether clinical criteria, including those for FM, are sufficient to discriminate those with active from those with inactive disease.

Recent work [5] has suggested that a tender joint count from 28 (TJC) minus swollen joint count (SJC) of at least 7 (joint count criteria) predicts with high sensitivity and specificity RA patients who also meet 1990 ACR FM classification criteria (tender point criteria). It is currently unknown whether the joint count or tender point count can differentiate patients with RA with genuinely lower inflammatory disease activity and hence be used to guide treatment decisions.

The aim of this pilot study was therefore to determine if RA patients with similar clinical disease activity assessed by DAS28 with clinical features of FM, as defined by the tender joint criteria have lower levels of joint inflammation compared with RA patients without FM as determined by US.

## Methods

### Participants

Patients meeting either the 1987 ACR or the 2010 ACR/EULAR 2010 RA classification criteria with DAS28(ESR) > 2.6 were recruited. Patients were categorized into those meeting or not meeting the joint count criteria i.e., TJC minus SJC ≥7, or TJC minus SJC < 7. Patients were recruited sequentially until approximately equal numbers were recruited to each group.

### Ethical approval

Research ethics committee approval was obtained from the Greenwich REC (# 234567) prior to commencing the study. The study was carried out according to the principles of Good Clinical Practice. All participants provided written informed consent.

### Assessments

Patients underwent clinical assessment including 28 tender/swollen joint count with ESR (DAS28) and assessment of soft-tissue tender points. The examining physician recorded the Symptom Severity Score (SSS). Patients completed a series of questionnaires including PHQ9 (depression), GAD7 (anxiety), PHQ15 (somatisation), FACIT-fatigue (fatigue), HAQ (disability), global assessment visual analogue scale, and the Widespread Pain Index (WPI).

Patients underwent ultrasound examination by a second physician blinded to the results of the clinical assessment. US was carried out in a darkened room, in a seated position with hands prone. Scanning was performed using Logic 9 (GE Healthcare) scanner with an 14 MHz transducer. US views were taken using standardised transducer orientation, taking longitudinal images of the MCPs, PIPs, and wrists (radiocarpal, ulnar carpal and intercarpal). PRF was set at 1.4KHz and PD gain was set to just below the threshold where PD signal disappeared. Grey scale (GS) still images and 3 s PD images were recorded. A semi quantitative score was used to grade GS and PD for each joint (0 no GS or PD signal, 1 = minimal, 2 = moderate, 3 = severe) according to validated criteria [5] and a total derived for each patient for each of GS and PD.

### Analysis

Statistical analysis was carried out using SPSS version 22 (IBM). Descriptive statistics (mean, standard deviation and confidence intervals) were used for patient characteristics. PD and GS ultrasound score differences between groups were tested using the Mann–Whitney *U* test. Significance was set at *p <* 0.05. Mann–Whitney U tests were used to compare difference between patient reported outcome measures between the two groups.

## Results

Forty-seven patients with active RA on disease-modifying therapy were recruited. The patient characteristics are shown in Table 1. The mean age of the patients was 61; 81 % were women, and 70 % were positive for rheumatoid factor. Mean DAS28 of the patients was 4.5, with 43 % with moderate disease activity (DAS28 > 3.2 and <5.1), and 36 % with high disease activity (DAS28 > 5.1). The mean HAQ was 1.5 (range 0.25-2.5).

**Table 1:** Patient characteristics

	TJC minus SJC ≥7 (TJC criteria)	TJC minus SJC <7	*P*	TP ≥11 (ACR 1990 criteria)	TP <11	*P*	Fulfil both ACR 1990 and TJC criteria	Do not meet both TJC and ACR criteria	*P*
	*n =* 26	*n =* 21		*n =* 19	*n =* 28		*n =* 17	*n =* 30	
Age (SD)	57	66		57	63		57	63	
Sex F (%)	22 (85)	16 (76)		16 (84)	22 (79)		15 (88)	24 (80)	
RF^a^ (%)	16 (62)	17 (81)		10 (53)	23 (82)		9 (53)	24 (80)	
DAS28 (SD)	4.99 (0.97)	4.03 (1.00)	0.002	5.23 (0.75)	4.11 (1.00)	<0.001	5.27 (0.68)	4.16 (1.07)	<0.001
TJ (SD)	15.15 (5.21)	5.24 (4.17)	<0.001	15.663 (5.84)	7.39 (5.40)	<0.001	16.47 (5.52)	7.47 (5.26)	0.002
SJ (SD)	1.50 (1.99)	3.52 (3.82)	0.67	1.84 (2.29)	2.79 (3.51)	0.44	1.71 (2.14)	2.80 (3.48)	0.457
ESR (SD)	18.96 (14.79)	20.67 (18.53)	0.94	20.53 (14.44)	19.18 (17.84)	0.38	18.35 (10.99)	20.50 (18.92)	0.157
PG (SD)	56.42 (18.45)	47.76 (20.82)	0.13	57.05 (18.31)	49.50 (20.52)	0.23	59.65 (17.50)	48.53 (20.18)	0.487

^a^missing data for one patient

Figures given as means and SD

*DAS28* twenty-eight joint disease activity score, *SJC* swollen joint count, *SJ* swollen joints, *TP* tender points, *TJC* tender joint count, *TJ* tender joints, *PG* patient global, *RF* rheumatoid factor

Forty percent of patients fulfilled the ACR 1990 Classification Criteria (CC) and/or the 2010 preliminary diagnostic criteria (DC) for fibromyalgia with 25 % meeting both criteria. 53 % of patients had TJC minus SJC ≥ 7 thus meeting the ‘joint count’ criteria and 36 % of patients met both ‘joint count’ and ‘classification’ criteria.

Mean DAS28 scores were significantly higher for patients meeting versus those not meeting FM classification criteria, with scores of 5.23 (SD 0.75) and 4.11 (1.0) respectively (*p <* 0.001). Mean DAS28 scores were also significantly higher for patients meeting versus not meeting the tender joint count with scores of 4.99 (0.97) and 4.03 (1.0) respectively (*p =* 0.002). Tender joint counts were also significantly higher in patients meeting the FM classification criteria versus those not meeting criteria 15.66 (5.84) and 7.39 (5.40) respectively (*p <* 0.001). Tender joint counts were also higher in patients meeting the tender joint count criteria versus those not meeting criteria with mean tender joint counts of 15.15 (5.21) and 5.24 (4.17) respectively. Patient global scores were numerically higher for patients meeting FM classification criteria versus those not meeting criteria with scores of 57.05 (18.31) and 49.50 (20.52) respectively, however these did not reach significance (*p =* 0.23). The same was true for patient global scores for patients meeting versus not meeting the tender joint criteria with scores of 56.42 (18.45) and 47.76 (20.82)(*p =* 0.13). There were no significant differences in swollen joint counts or ESR. These results are shown in Table 1.

GS US scores were significantly lower in patients meeting either the FM classification or joint count criteria and for patients that fulfilled both criteria. PDUS scores for patients meeting either of these criteria were numerically lower but did not meet significance. However, patients who met both FM classification and joint count criteria had significantly lower PDUS scores (2.94, FM group vs 8.33, non-fibromyalgic group, *p =* 0.028) than those meeting a single fibromyalgia criteria or none. When the 2010 ACR preliminary diagnostic FM criteria were used, no differences were seen in US scores or other objective or subjective clinical measures except higher DAS28 scores and tender joint counts.

Significantly higher levels of fatigue (GAD7), depression (PHQ9) and disability (HAQ) were also seen in patients meeting either the joint count or classification criteria (Table 2). Scores for somatic symptoms and fatigue were not significantly different.

**Table 2:** Patient reported outcome measures: FACIT-fatigue (FF), GAD7 (anxiety), Health Assessment Questionnaire (HAQ), PHQ9 (depression), PHQ15 (somatization), Symptom Severity Score (SSS), Widespread Pain Index (WPI)

	TJC minus SJC ≥7 (joint count criteria)	TJC minus SJC <7	*P*	TP ≥11 (ACR 1990 classification criteria)	TP <11	*P*	Fulfil both ACR 1990 and joint count criteria	Do not meet both joint count and ACR criteria	*P*
	*n =* 26	*n =* 21		*n =* 19	*n =* 28		*n =* 17	*n =* 30	
GAD7	7.58	3.62	0.005	7.84	4.43	0.001	7.71	4.73	0.020
PHQ9	9.27	4.52	0.017	10.89	4.61	0.000	10.59	5.20	0.027
PHQ15	10.77	6.90	0.019	11.79	7.18	0.226	11.88	7.43	0.230
HAQ	1.70	1.27	0.070	1.84	1.29	0.002	1.85	1.32	0.005
FF	25.85	15.81	0.461	27.47	17.21	0.332	28.29	17.43	0.725
WPI	7.15	4.81	0.145	9.47	4.25	0.073	9.41	4.62	0.886
SSS	7.50	4.95	0.699	7.53	5.14	0.316	7.65	5.23	0.494

*SJC* swollen joint count, *TJC* tender joint count, *TP* tender points

## Discussion

Around 20 % of patients with RA may have concomitant fibromyalgia [3]. A number of studies have shown that RA patients with comorbid fibromyalgia, in most cases as defined as widespread pain with soft tissue tenderness, tend to have higher disease activity scores despite less objective evidence of active inflammatory disease [4]. This suggests that conventional disease activity scores may not be able to differentiate patients with differing causes of high DAS28 scores to allow selection of optimal treatment strategies. Patients with generalized pain driven by non-inflammatory mechanisms are unlikely to respond to therapies focused on suppressing inflammation. In our study median DAS28 scores were higher in patients meeting all definitions of fibromyalgia, an effect mainly driven by differences in the tender joint count.

Is has been reported that a TJC minus SJC score greater than ≥ 7 (‘joint count’ criteria) identifies RA patients who are more likely to meet the 1990 ACR classification criteria for fibromyalgia, who score highly for disability, depression and fatigue [3]. This is supported by further data showing psychological distress and poor quality sleep, reduce pain thresholds in people with RA [3]. Psychological distress is predictive of the development of FM in patients with early inflammatory arthritis [6, 7]. Within the group of RA patients with concomitant FM and active disease defined by DAS28, but limited objective evidence of joint inflammation, it is unclear whether clinical measures can differentiate those with active synovial inflammation from those without.

PDUS is more sensitive than clinical examination for the presence of inflammation [8]: it is predictive of outcomes in RA and is hence increasingly used in clinical practice to inform treatment decisions [9]. It therefore has the potential to help to differentiate patients with joint pain secondary to generalised widespread pain from those with active synovial inflammation.

In this study we did not find a significant difference in PDUS scores between the FM versus non FM patients when the tender joint criteria were used. It is well-established that active synovitis can be demonstrated by PD in joints which are not clinically swollen [10] and so this may not be surprising. Furthermore, no significant differences were seen when either the 1990 classification or 2010 diagnostic criteria were used alone. Neither of the latter have been validated in the RA patient population. The identification of widespread non-inflammatory pain does not preclude the presence of active synovial inflammation so this is not an unexpected finding. A recent study comparing GS and PDUS findings in RA patients meeting or not meeting the 2010 diagnostic criteria for FM found no difference in total GSUS or PDUS scores of a panel of 7 joints [11]. The diagnostic criteria for FM include assessment of fatigue, cognitive symptoms, sleep quality and somatization. However, RA patients score higher for poor sleep quality, fatigue, cognitive and somatic symptoms than controls [12, 13], which are predictive of worse symptoms overall in RA patients regardless of the presence of FM [14]. It is therefore not surprising that the FM diagnostic criteria alone lack sensitivity for FM in a population with RA. Our findings support the view that the DAS28 may lack sensitivity for inflammatory activity where there is little or no joint swelling, and therefore there may be a role for the use of US to guide treatment decisions.

We showed that FM classification criteria or joint count criteria alone did not differentiate patients with higher vs. lower PDUS scores, but when combined they were able to differentiate patients with lower ultrasound scores. This supports our suggestion that more rigorous composite criteria, defining both the presence of widespread pain and limited clinical inflammatory disease activity, are necessary to differentiate patients with lower US scores. Our findings that RA patients meeting the FM classification or joint count criteria scored more highly for depression, fatigue, somatic symptoms and disability, replicates that of previous studies. We suggest that patients with fibromyalgia, RA and high DAS28 scores could be identified by more a comprehensive assessment incorporating clinical examination, serology and patient-reported outcome measures.

More accurate stratification of the subgroup of patients with RA and non-inflammatory widespread pain could help optimise treatment decisions. If the causes of symptoms and signs in this difficult to define group can be clearly identified, it will allow the increasingly useful therapies for fibromyalgia or inflammatory arthritis to be used with greater precision [15]. Importantly, therapies for FM are also effective in RA patients [16] and these strategies may thus help more sophisticated stratification of patients into different treatment regimens.

Our study has several limitations. Only the hands and wrists were assessed by US whereas inclusion of other joints may provide additional information. It is unclear how many and which joints should be included and it may be that a larger panel of joints would have produced different results. The number of patients included in this study was relatively small and these findings need to be replicated in a bigger sample. A larger study is underway in our centre to determine whether multiple clinical measures of disease activity and patient-reported measures of other factors which may be associated with widespread non-inflammatory pain can better differentiate these groups.

## Conclusion

This preliminary study has shown that composite clinical tools may help to differentiate patients with RA and concomitant fibromyalgia with DAS28 > 2.6 who have lower ultrasonographic disease activity. These patients are less likely to respond to escalation of inflammation-suppressing therapy and may be more suitable for other forms of treatment including alternative means of pain control and psychological support. Further work is needed to determine whether clinical criteria can be used as predictive tools to identify these patients or whether imaging is necessary for accurate stratification. We are currently undertaking a larger study exploring correlation between multiple clinical parameters and ultrasonographic measures of inflammation.

## Abbreviations

DAS28: 28 joint disease activity score
FM: Fibromyalgia
FMRA: Fibromyalgic rheumatoid arthritis
GS: Grey scale
HAQ: Health Assessment Questionnaire
MCP: Metacarpo-phalangeal joint
PD: Power Doppler
PG: Patient global
PIP: Proximal interphalangeal joint
RA: Rheumatoid arthritis
SJC: Swollen joint count
SSS: Symptom severity scale
TJC: Tender joint count
WPI: Widespread pain index
